# A novel fatty acid-binding protein 5-estrogen-related receptor α signaling pathway promotes cell growth and energy metabolism in prostate cancer cells

**DOI:** 10.18632/oncotarget.25878

**Published:** 2018-08-03

**Authors:** Shogo Senga, Koichiro Kawaguchi, Narumi Kobayashi, Akira Ando, Hiroshi Fujii

**Affiliations:** ^1^ Interdisciplinary Graduate School of Science and Technology, Shinshu University, Minami-minowa, Kami-ina, Nagano, 399-4598, Japan; ^2^ Department of Biomedical Engineering, Graduate School of Science and Technology, Shinshu University, Minami-minowa, Kami-ina, Nagano, 399-4598, Japan; ^3^ Department of Interdisciplinary Genome Sciences and Cell Metabolism, Institute for Biomedical Sciences, Interdisciplinary Cluster for Cutting-Edge Research, Shinshu University, Minami-minowa, Kami-ina, Nagano, 399-4598, Japan

**Keywords:** FABP5, ERRα, PGC-1β, energy metabolism, prostate cancer

## Abstract

Epidermal or cutaneous fatty acid-binding protein is an intracellular lipid-binding protein, also known as FABP5, and its expression level is closely related to cancer cell proliferation and metastatic activities in various types of carcinoma. However, the molecular mechanisms of FABP5 in cancer cell proliferation and its other functions have remained unclear. In the present study, we have clearly revealed that FABP5 activated expression of metabolic genes (ATP5B, LCHAD, ACO2, FH and MFN2) via a novel signaling pathway in an ERRα (estrogen-related receptor α)-dependent manner in prostate cancer cell lines. To clarify the novel function of FABP5, we examined the activation mechanisms of the ERRα target genes via FABP5. A direct protein-protein interaction between FABP5 and ERRα was demonstrated by immunoprecipitation and GST pull-down assays. We have clearly revealed that FABP5 interacted directly with transcriptional complex containing ERRα and its co-activator PGC-1β to increase expression of the ERRα target genes. In addition, we have shown that FABP5 knockdown induced high energy stress leading to induction of apoptosis and cell cycle arrest via AMPK-FOXO3A signaling pathway in prostate cancer cells, suggesting that FABP5 plays an important role in cellular energy status directing metabolic adaptation to support cellular proliferation and survival.

## INTRODUCTION

Prostate cancer (PCa) is the most common cancer in men worldwide and the second leading cause of male cancer-related deaths in the United States [1, 2]. The onset of PCa is associated with a high fat diet and aberrant lipid metabolism induced by systemic metabolic diseases, but the precise molecular mechanism of PCa remains unclear [3, 4]. An increase in de novo lipogenesis is observed and is significantly associated with tumor progression in various tissues, including prostate [5–7]. In addition, lower rates of glycolysis and higher rates of fatty acid oxidation have been characterized in PCa cells. Therefore, understanding the regulation of fatty acid metabolism in PCa is important for developing novel therapeutic strategies and diagnostic tools.

The fatty acid-binding proteins (FABPs) are a family of low-molecular weight, intracellular lipid-binding proteins consisting of ten isoforms. FABPs are responsible for uptake and transport of fatty acid [8, 9]. Recent studies have reported that FABPs play important roles in the regulation of gene expression, cell growth and differentiation [10, 11]. Multiple reports have demonstrated that metabolic reprogramming is necessary to sustain cancer cell growth and survival [12–14]. Alteration in fatty acid metabolism is a hallmark of cancer, especially in PCa cells, and several lines of evidence showed that limiting fatty acid availability controls cancer cell proliferation [15–17]. Since fatty acids are required as an energy source, the formation of membrane components, and the production of cellular signaling molecules during cancer cell proliferation, FABPs might play an important role in cell proliferation in cancer cells [15].

We recently reported that among the ten FABPs, FABP5 was specifically upregulated via epigenetic mechanisms during carcinogenesis [18] and promoted tumorigenesis in PCa cells [18–24]. FABP5 is also increased in various cancers, including triple negative breast cancer [25, 26], bladder cancer [27], pancreatic cancer [28], oral squamous cell carcinoma [29], and intrahepatic cholangiocarcinoma [30]. We also showed that FABP5 regulated the expression of metabolic genes via an PPARβ/δ (peroxisome-proliferator-activated receptor β/δ)-independent pathway in colorectal cancer cells [31]. However, the molecular mechanisms remain unclear.

In this study, we hypothesized that FABP5 also exhibits PPARs-independent functions. Here we examined and revealed the mechanisms of FABP5 in cancer cell proliferation and metabolism in PCa via a PPARβ/δ-independent pathway.

## RESULTS

### High expression of FABP5 promotes cell growth in various PCa cells depending on their malignancy

**Figure 1:**
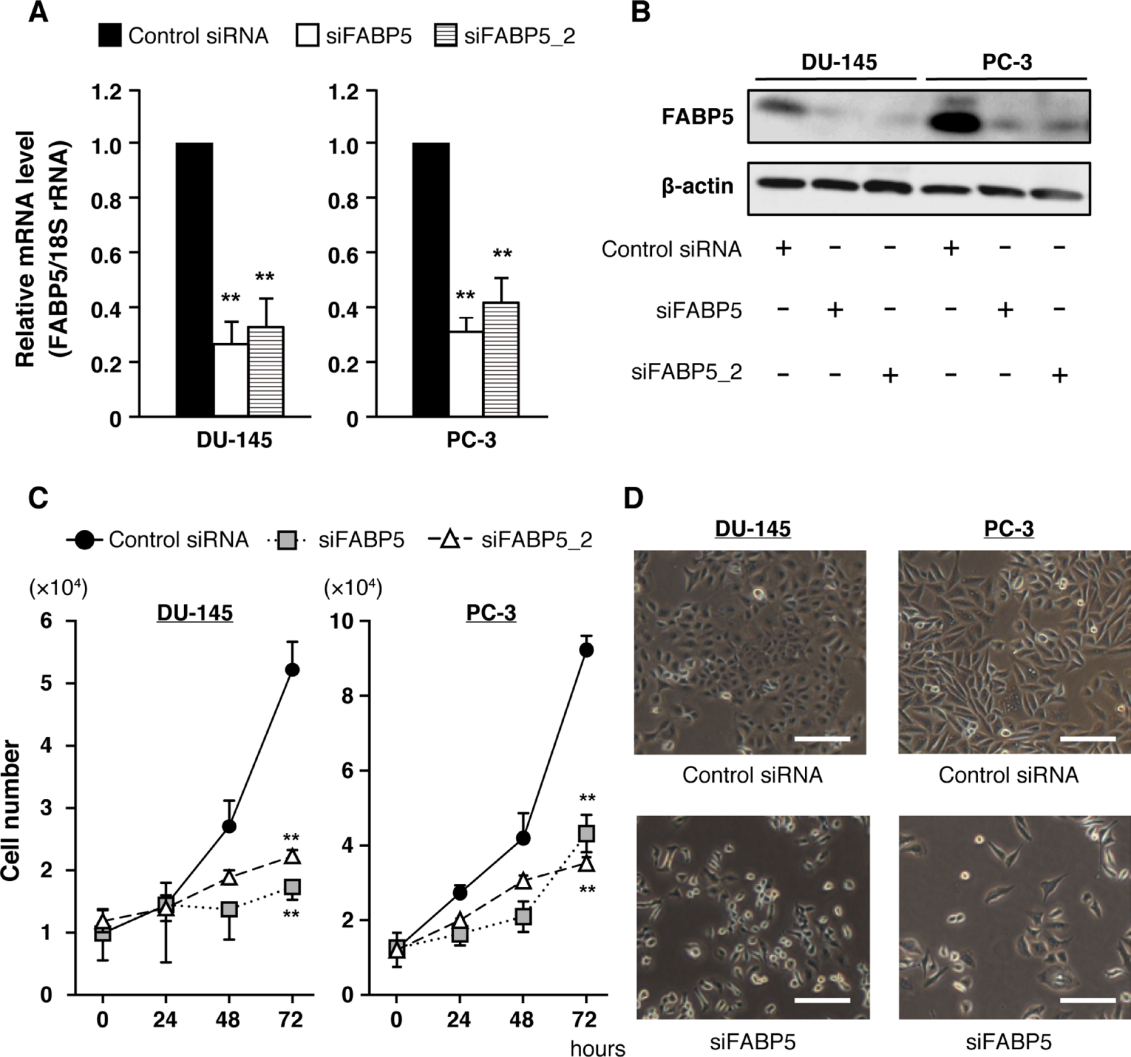
FABP5 knockdown significantly suppresses cell growth in PCa cells (**A**) FABP5 mRNA levels in PCa cells transfected with control siRNA (20 nM) or siFABP5 (20 nM) determined by qPCR. Results are means ± S.D. for three independent experiments. (**B**) Western blot analysis of FABP5 in PCa cells transfected with control siRNA (20 nM) or siFABP5 (20 nM). β-actin served as an internal loading control. Results shown are representative of three independent experiments. (**C**) Cell proliferation of control siRNA (20 nM) or siFABP5 (20 nM) transfected PCa cells. Cells were counted at the indicated times. Results are means ± S.D. for three independent experiments. (**D**) Representative images of cells transfected with control siRNA (upper panel) or siFABP5 (lower panel) 72 h after transfection. Scale bar, 200 μm. ^**^P < 0.01, one-way ANOVA followed by Dunnett's test (A), two-way ANOVA followed by Tukey's test (C).

We first examined FABP5 mRNA expression in various PCa cell lines and found that FABP5 was strongly expressed in DU-145, PC-3 and PC-3M (malignant cancer cell lines) (Supplementary Figure 1A). Previous studies showed that FABP5 promotes cell proliferation of cancer cells [22, 23, 25, 29–31]. Therefore, we examined whether FABP5 knockdown would affect cell proliferation of PCa cells (DU-145, PC-3 and PC-3M) (Figure 1A, 1B and Supplementary Figure 1B, 1C). FABP5 knockdown induced significant suppression of cell growth in PCa cells (Figure 1C, 1D and Supplementary Figure 1D, 1E). In addition, compensatory effects by other FABPs might not occur in this experimental condition, because the expression levels of other FABPs were not affected by FABP5 knockdown (Supplementary Figure 1F). These results suggest that FABP5 plays an important role in promotion of cancer cell proliferation. However, the molecular mechanisms underlying the connection between high expression of FABP5 and cancer cell proliferation remains unclear. Thus, we next investigated how FABP5 promoted cell proliferation in PCa cells.

### Nuclear localization of FABP5 promotes PCa cell proliferation

**Figure 2:**
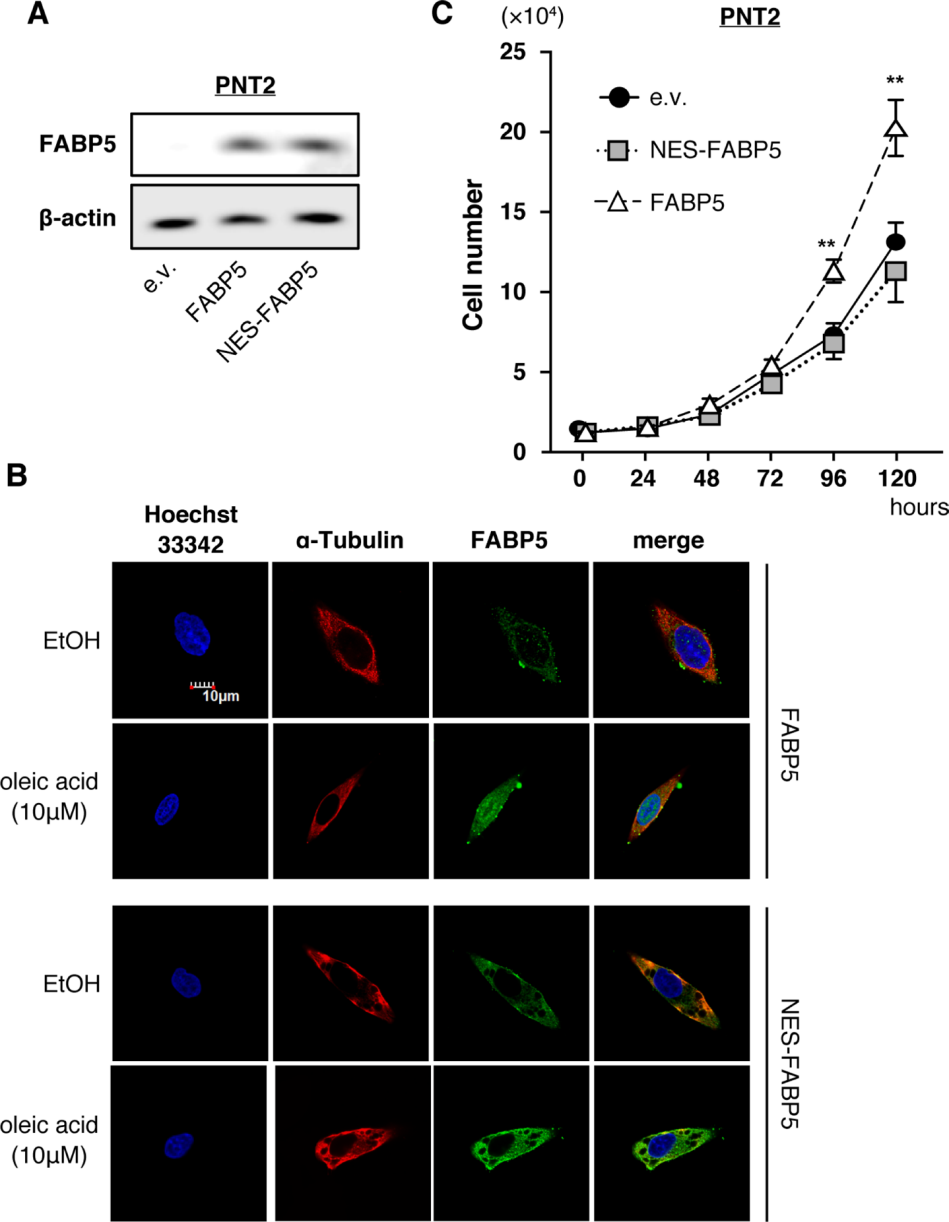
Nuclear localization of FABP5 promotes cell proliferation (**A**) Western blot analysis of FABP5 and NES-FABP5 in PNT2 cells transfected with pCI-neo/FABP5, NES-FABP5 or pCI-neo (e.v., empty vector). Results shown are representative of three independent experiments. (**B**) Localization of FABP5 and NES-FABP5 in FABP5 or NES-FABP5 overexpressing PNT2 cells. Transfected cells were treated with ethanol or oleic acid (10 μM) for 30 min. (**C**) Cell growth of FABP5- and NES-FABP5-transfected PNT2 cells. Cells were counted at the indicated times. Results are means ± S.D. for three independent experiments. ^**^P < 0.01, two-way ANOVA followed by Tukey's test.

To investigate how FABP5 overexpression can lead to cell proliferation in PCa, we investigated its localization in PCa cells. FABP5 relocalization from the cytoplasm to the nucleus is dependent upon its binding to a ligand (fatty acid), and nuclear localization plays a critical role in its functions [32]. Therefore, we examined whether the cell proliferation functions mediated by FABP5 were induced in the nucleus or the cytoplasm. We generated a nuclear export signal-tagged FABP5 expression plasmid (NES-FABP5, Supplementary Figure 2A), similar to a previous study [33]. NES-FABP5 was able to bind to ligands (fatty acid) as well as FABP5 in fluorescence-based binding assays using the fatty acid analog DAUDA (11-(dansylamino)undecanoic acid) (Supplementary Figure 2B and 2C). We confirmed relatively equal expression of FABP5 and NES-FABP5 in transfected PNT2 cells (normal prostate cells) (Figure 2A) and next compared their physiological functions. FABP5 was redistributed in response to oleic acid from the cytoplasm to the nucleus, while NES-FABP5 remained in the cytoplasm (Figure 2B). Notably, FABP5 overexpression significantly promoted cell proliferation in PNT2 cells, whereas no such promotion was observed in NES-FABP5-overexpressing PNT2 cells (Figure 2C). These results strongly suggested that nuclear localization of FABP5 was required for promoting PCa cell proliferation.

### FABP5 contributes to PCa cell proliferation via PPARβ/δ-independent pathway

**Figure 3:**
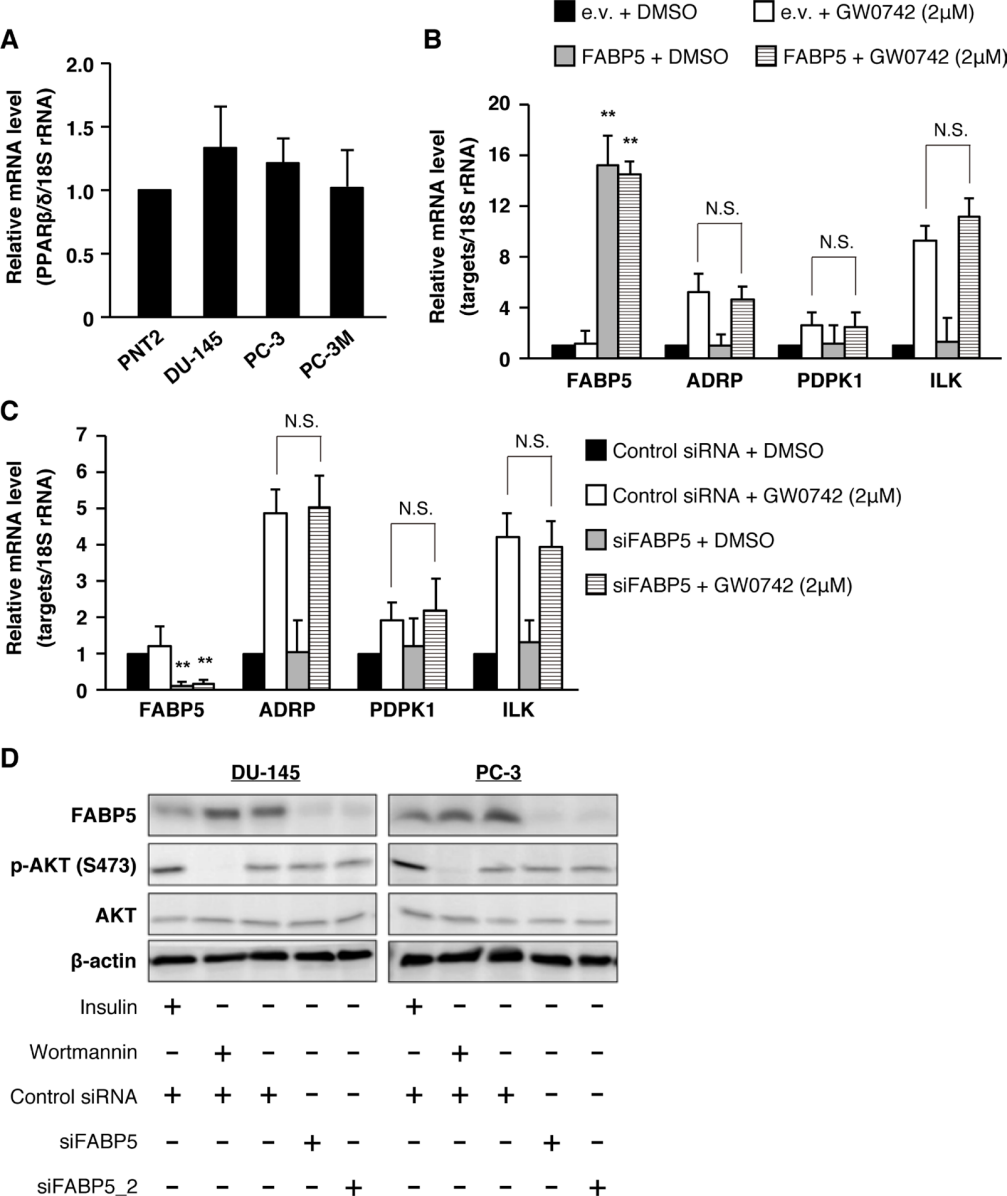
PPARβ/δ signaling might not be involved in FABP5-mediated growth promotion of PCa cells (**A**) PPARβ/δ mRNA expression levels in PNT2, DU-145, PC-3 and PC-3M. Relative mRNA levels were measured by qPCR. Results are means ± S.D. for three independent experiments. (**B**) PNT2 cells were transfected with pCI-neo or pCI-neo/FABP5. At 48 h after transfection, transfected cells were treated with DMSO or GW0742 (2 μM) for 24 h. mRNA levels of FABP5, PDPK1, ADRP and ILK were measured by qPCR and normalized to those of 18S rRNA. Results are means ± S.D. for three independent experiments. (**C**) PC-3 cells were transfected with control siRNA (20 nM) or siFABP5 (20 nM). At 48 h after transfection, cells were treated with DMSO or GW0742 (2 μM) for 24 h. FABP5, PDPK1, ADRP and ILK mRNA levels were measured by qPCR and normalized to those of 18S rRNA. Results are means ± S.D. for three independent experiments. (**D**) Phosphorylation of Akt (S473) in siFABP5 transfected DU-145 and PC-3 was detected by western blot analysis. We used insulin (as positive control of AKT phosphorylation, 1 μg/ml, 15 min) and wortmannin (inhibitor of phosphoinositide 3-kinases, as negative control of AKT phosphorylation, 0.5 μM, 30 min). Results shown are representative of three independent experiments. ^**^P < 0.01, one-way ANOVA followed by Tukey's test (B and C), N.S. = Not significant.

Previous studies suggested that FABP5 regulates the signaling activities of peroxisome proliferator-activated receptor β/δ (PPARβ/δ), a ligand-dependent transcription factor, by functioning as a free fatty-acid transporter in various cancer cells [32, 34]. However, our recent study strongly suggested that FABP5 promoted cell growth and metastatic potency in colorectal cancer cells in a PPARβ/δ-independent manner [31]. To investigate the relationship between PPARβ/δ activity and FABP5 in PCa cells, we analyzed expression levels of PPARβ/δ target genes, 3-phosphoinositide-dependent protein kinase-1 (PDPK1), Adipose differentiation-related protein (ADRP) and Integrin-linked kinase (ILK), in FABP5 overexpressed PNT2 cells with/without highly selective PPARβ/δ agonist GW0742, which can also bind to FABP5 [32]. PPARβ/δ was abundantly expressed at the same level in PNT2 cells as malignant PCa cells (Figure 3A). PDPK1, ADRP and ILK mRNA levels were increased by GW0742. Strikingly, however, these gene levels were not elevated by FABP5 overexpression (Figure 3B). We next assessed expression levels of the PPARβ/δ target genes in PCa cells when FABP5 expression was depleted by siRNA. FABP5 knockdown did not significantly affect expression of the PPARβ/δ target genes (Figure 3C and Supplementary Figure 3A), suggesting that PPARβ/δ-dependent transcriptional activities in PCa cells were not influenced by FABP5 levels.

Previous studies also showed that the FABP5-PPARβ/δ pathway involved in cell proliferation is associated with AKT phosphorylation in breast cancer cells [35]. Thus, we further analyzed whether FABP5 regulates AKT phosphorylation in PCa cells. Although PPARβ/δ was actually involved in regulation of PCa cell proliferation (Supplementary Figure 3B and 3C), FABP5 expression level had no impact on the phosphorylation level of AKT (Figure 3D), indicating that the FABP5-PPARβ/δ signaling axis might be physiologically inactive in PCa cells. These observations, thus, prompted us to further explore novel signaling pathways that may be involved in PCa cell proliferation mediated by FABP5.

### FABP5 is functionally associated with ERRα target genes in PCa cells

**Figure 4:**
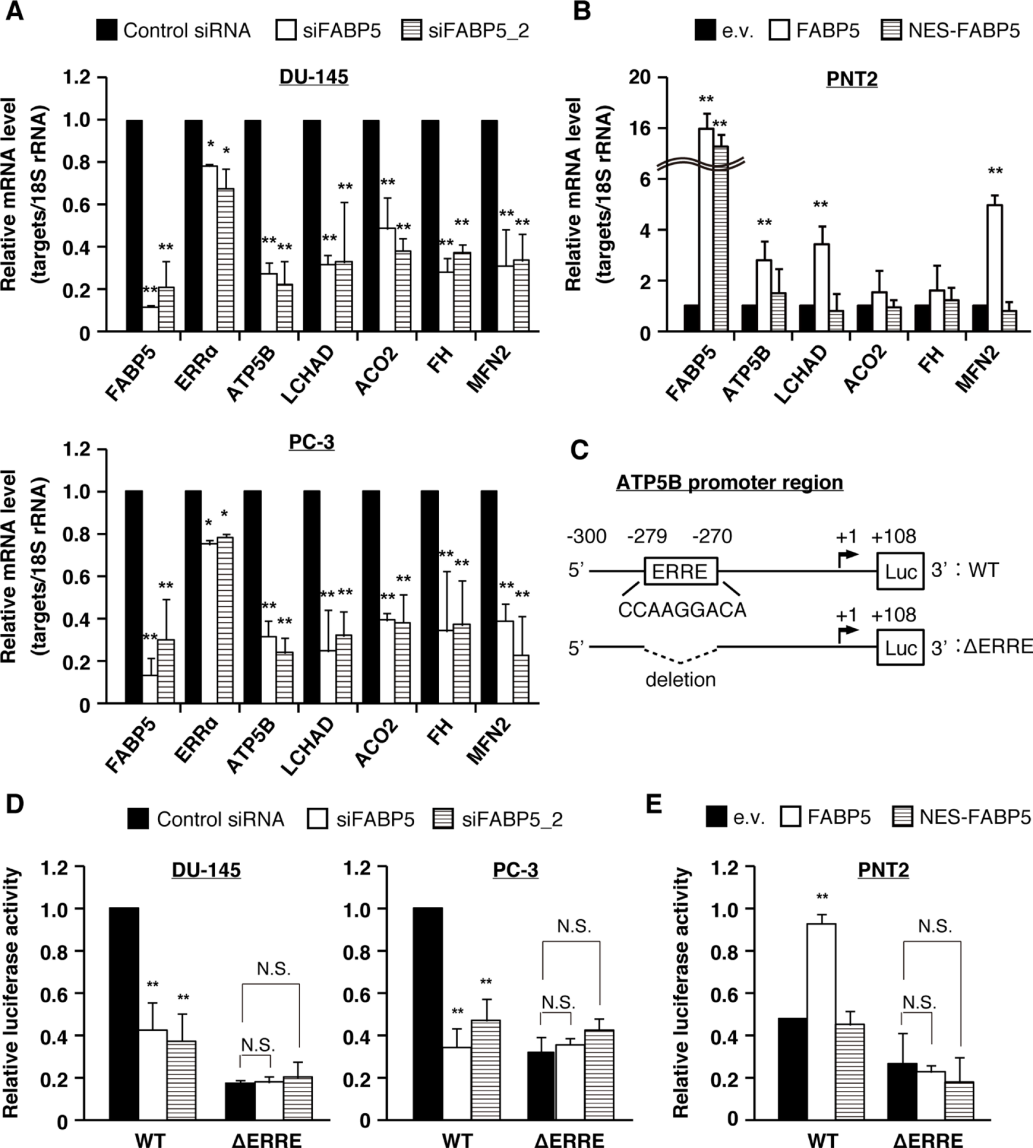
FABP5 promotes cell growth via ERRα signaling pathway in PCa cells (**A**) PCa cells were transfected with control siRNA (20 nM) or siFABP5 (20 nM) and mRNA levels of various factors were determined by qPCR. Results are means ± S.D. for three independent experiments. (**B**) PNT2 cells were transfected with pCI-neo (e.v.), pCI-neo/FABP5 (FABP5) or pCI-neo/NES-FABP5 (NES-FABP5) and mRNA levels of various factors were measured by qPCR. Results are means ± S.D. for three independent experiments. (**C**) Schematic of the ATP5B promoter region and luciferase constructs with the wild-type (WT) sequence or mutant deleted for the ERRE (ΔERRE). (**D**) The luciferase reporter vector driven by the ATP5B promoter (WT) or ΔERRE mutant was co-transfected with hRluc/TK into PC-3 and DU-145 cells, and treated with control siRNA (20 nM) or siFABP5 (20 nM). Luciferase activities were measured and normalized against Renilla activities. Results are means ± S.D. for three independent experiments. (**E**) The WT or ΔERRE mutant reporter vector was co-transfected with hRluc/TK into PNT2 cells transfected with pCI-neo (e.v.), pCI-neo/FABP5 or NES-FABP5. Luciferase activities were measured and normalized against Renilla activities. Results are means ± S.D. for three independent experiments. ^*^P < 0.05, ^**^P < 0.01, one-way ANOVA followed by Dunnett's test (A, B, D and E), N.S. = Not significant.

To examine which signaling pathways may be associated with FABP5 in PCa cells, we investigated the levels of various genes associated with apoptosis, cell cycle, metabolism, cell adhesion, stemness and other functions in PCa cells transfected with siFABP5. Many metabolic genes, especially target genes for estrogen-related receptor α (ERRα), were significantly decreased by FABP5 knockdown (ATP synthase subunit beta, ATP5B; long-chain 3-hydroxyacyl-CoA dehydrogenase, LCHAD; Aconitase 2, ACO2; Fumarate hydratase, FH; Mitofusin-2, MFN2) (Figure 4A and Supplementary Figure 4A). In addition, these target genes were increased by FABP5 overexpression in PNT2 cells (Figure 4B). ERRα is an orphan nuclear receptor associated with mitochondrial functions and somatic cell reprogramming [36, 37]. Recent studies revealed that ERRα has oncogenic functions in breast, ovarian, lung, colon and prostate cancers [38–45]. We found that ERRα target genes were scarcely decreased in PCa cells treated with GSK-3787 (Supplementary Figure 4B), whereas the genes were declined by ERRα suppression (Supplementary Figure 4C) as well as by NES-FABP5 expression, which antagonizes FABP5 functions in the nucleus (Supplementary Figure 4D). ERRα was highly expressed in normal and PCa cells (Supplementary Figure 4E), and cell growth of PCa cells was significantly suppressed by ERRα knockdown (Supplementary Figure 4F). In addition, the ERRα responsive element (ERRE), whose consensus sequence is TNAAGGTCA (where N is any nucleotide), is found in the promoter regions of genes regulating mitochondrial function [46]. We thus hypothesized that FABP5 localizes to the nucleus and regulates expression of these metabolic genes via ERRα activation.

To confirm this hypothesis, we examined whether FABP5 is involved in ERRα mediated gene expression by reporter gene assays in PCa cells. ATP5B is well characterized as an ERRα direct target gene and contains an ERRE in its promoter region [36]. We generated a luciferase reporter vector driven by the *ATP5B* promoter region containing the ERRE motif as well as a mutant reporter deleted for the ERRE (Figure 4C). Reporter activity in PCa cells was attenuated by knockdown of FABP5; in contrast, the mutant reporter vector showed lower reporter activity but knockdown of FABP5 had no impact on these levels (Figure 4D). Moreover, overexpression of FABP5 significantly increased WT reporter activity, but failed to increase the mutant reporter activity (Figure 4E). Importantly, NES-FABP5 could not promote ERRα-dependent luciferase activity. Consistent with this observation, ERRα target genes were increased by FABP5 overexpression in PNT2 cells, but were not by overexpression of NES-FABP5 (Figure 4B). Together, this suggests a crucial role of FABP5 in activating ERRα-dependent transcription.

### FABP5 directly interacts with ERRα/PGC-1β to activate target genes in PCa cells

**Figure 5:**
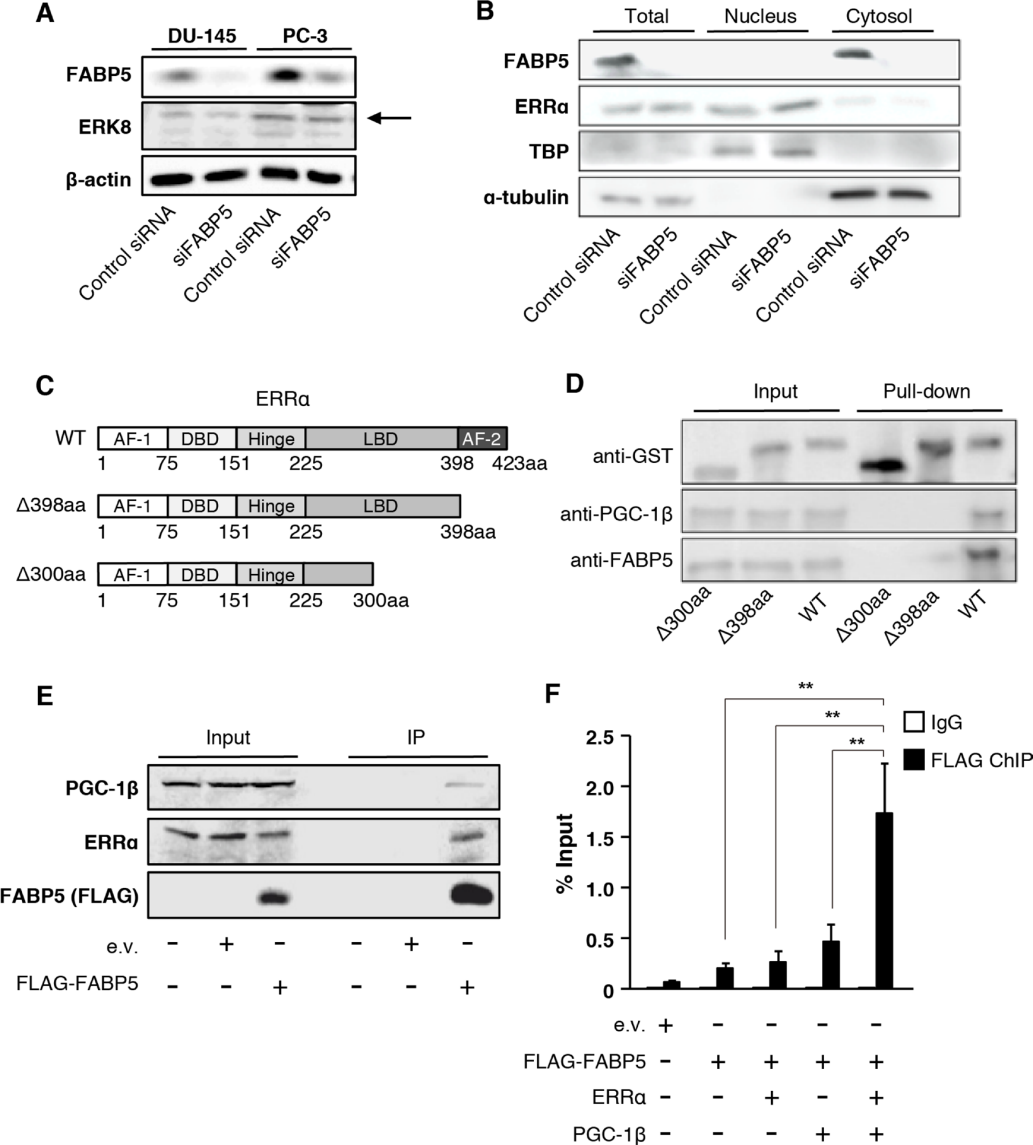
FABP5 activates ERRα target genes mediated by direct interaction with ERRα in the nucleus (**A**) Detection of ERK8 expression level in control siRNA (20 nM) or siFABP5 (20 nM) transfected PCa cells by western blot analysis. The arrow indicates ERK8 band. Results shown are representative of three independent experiments. (**B**) Western blot analysis of ERRα in nuclear and cytoplasmic fractions from control siRNA (20 nM) or siFABP5 (20 nM) transfected PC-3 cells. TBP served as a nuclear marker and tubulin served as cytoplasmic marker. (**C**, **D**) GST pull-down assays. ERRα contains an activation function 1 (AF-1, 1~75aa), a central zinc finger DNA binding domain (DBD, 75~151aa), a hinge region (151~225aa), a ligand-binding domain (LBD, 225~423aa), and an activation function 2 domain (AF-2, 398~423aa). We prepared two deletion mutants of ERRα (Δ300aa and Δ398aa). GST, GST-ERRα, GST-ERRα Δ300aa or GST-ERRα Δ398aa was incubated with PC-3 whole cell lysates and glutathione sepharose overnight at 4°C. GST, FABP5 and PGC-1β levels were detected by western blot analysis. Results shown are representative of three independent experiments. (**E**) Immunoprecipitation was performed with FLAG antibody (MBL) on lysates from pCI-neo (e.v.) or pCI-neo/3×FLAG-FABP5-transfected 293T cells. Input (10%) and IP samples were analyzed by western blot analysis. Results shown are representative of three independent experiments. (**F**) ChIP–qPCR analysis to evaluate binding of the FABP5-ERRα complex to the ATP5B promoter. 293T cells were transfected with pCI-neo (e.v.), FLAG-FABP5, ERRα and pGC-1β as indicated. ChIP was performed using anti-DDDDK-tag antibody, and the ERRα-binding site in ATP5B (−300 to −213) promoter region was amplified using ChIP-qPCR primers to calculate % input. Primer sequences are described in the SI Materials and Methods. Results are mean ± S.D. for three independent experiments. ^**^P < 0.01, one-way ANOVA followed by Tukey's test.

We next investigated the molecular mechanisms underlying the FABP5-ERRα crosstalk. A previous study showed that the cellular localization of ERRα is regulated by extracellular signal-regulated kinase 8 (ERK8), which directly transports ERRα from the nucleus to the cytoplasm [47]. Therefore, we analyzed ERK8 expression levels and ERRα localization in FABP5 knockdowned PCa cells. Immunoblot analyses revealed that both ERK8 expression levels (Figure 5A and Supplementary Figure 5A) and cellular localization of ERRα (Figure 5B and Supplementary Figure 5B) were not affected by FABP5 knockdown.

We hypothesized that FABP5 might interact with the transcriptional complex including ERRα in the nucleus and increase transcriptional activities of target genes. To test the physical interaction between FABP5 and ERRα, we performed *in vitro* GST pull-down assays using a GST-ERRα fusion protein and PCa (PC-3) cell lysates (Figure 5C and 5D). To determine the FABP5 binding site in ERRα, we constructed GST fusion ERRα deletion mutants (WT, Δ300aa and Δ398aa) (Figure 5C). GST-ERRα effectively bound endogenous FABP5 (Figure 5D). We also observed that peroxisome proliferator-activated receptor gamma coactivator 1-beta (PGC-1β), a transcriptional co-regulator of ERRs, was pulled down with GST-ERRα, suggesting the possibility that FABP5 might be recruited to the transcriptional complex. The mutant that lacks the activation function 2 domain (AF-2) of ERRα (Δ398aa) weakly interacted with FABP5, and that lacks the ligand-binding domain (LBD) of ERRα (Δ300aa) could not bind to FABP5 and PGC-1β. These results suggested that FABP5 specifically associates with ERRα/PGC-1β complex via the carboxyl terminal domain containing the half of LBD and AF-2 of ERRα (Figure 5D). Especially, AF-2 domain might be important to form complex.

Moreover, we performed immunoprecipitation assays to confirm physiological and physical interaction. Immunoprecipitation assays demonstrated that FABP5 and endogenous ERRα, as well as PGC-1β, were co-immunoprecipitated in 293T cells (Figure 5E). In this experiment, FABP5/ERRα/PGC-1β complex might include other factors that promote the formation of this complex. At least, FABP5 could interact with ERRα/PGC-1β in the physiological condition.

We next examined whether this complex could form physiological interactions with DNA sequences in the ATP5B promoter using chromatin immunoprecipitation (ChIP) assays followed by qPCR. We showed that ATP5B was regulated by FABP5 via ERRα activation (Figure 4C–4E). As predicted, binding of FABP5 was detected at the promoter region of ATP5b (Figure 5F). Notably, co-transfection with ERRα and PGC-1β increased FABP5 recruitment to the ATP5B promoter, indicating FABP5/ERRα/PGC-1β crosstalk *in vitro*. Collectively, these results strongly suggest that FABP5 directly functions as a potent transcriptional cofactor to regulate ERRα-dependent transcriptional activity in PCa cells.

### FABP5 suppression induced G1 cell cycle arrest and apoptosis via the AMPK-FOXO3A pathway in PCa cells

**Figure 6:**
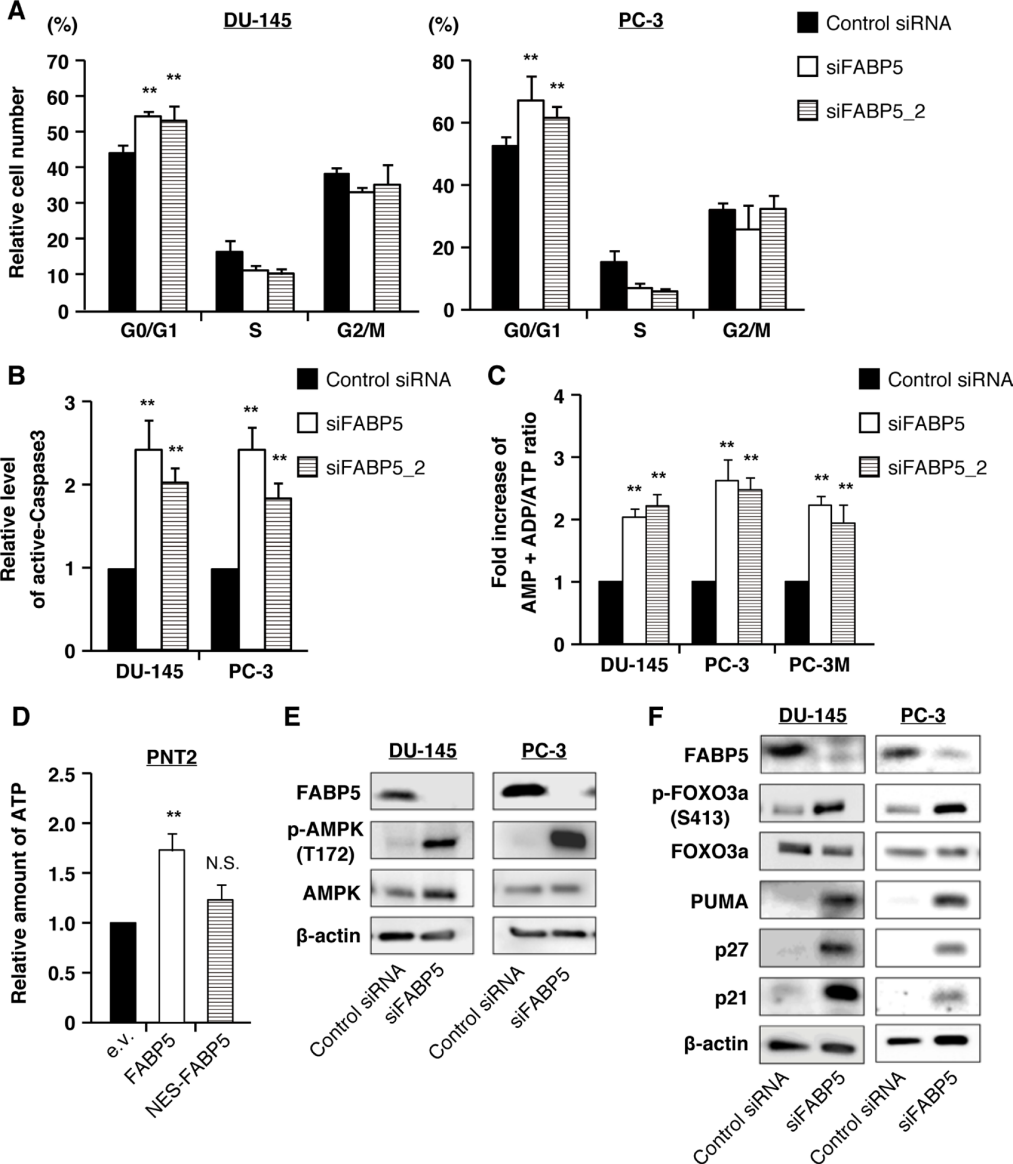
FABP5 knockdown induces starvation stress and activation of the AMPK-FOXO3A signaling pathway in PCa cell lines (**A**) Cell cycle analysis of control siRNA (20 nM) or siFABP5 (20 nM) transfected PCa cells by flow cytometry. Percentages of cells at each phase are indicated. (**B**) Apoptosis was measured by cleaved caspase 3 (active form) level by flow cytometry. (**C**) Amounts of AMP+ADP and ATP were measured by the microplate reader using firefly luciferase in control siRNA (20 nM) or siFABP5 (20 nM) transfected PCa cells. Data are presented as the means ± SD from four independent experiments. (**D**) Relative amount of ATP was measured using firefly luciferase in pCI-neo (e.v.), pCI-neo/FABP5 or pCI-neo/NES-FABP5-transfected PNT2 cells. Results are mean ± S.D. for twenty independent experiments. (**E**) Western blot analysis of AMPK and phosphorylated AMPK in prostate cancer cells transfected with control siRNA (20 nM) or siFABP5 (20 nM). Results shown are representative of three independent experiments. (**F**) Western blot analysis of FOXO3A and proteins encoded by its target genes in PCa cells transfected with control siRNA (20 nM) or siFABP5 (20 nM). Results shown are representative of three independent experiments. ^**^P < 0.01, two-way ANOVA followed by Tukey's test (A), one-way ANOVA followed by Dunnett's test (B, C and D), N.S. = Not significant.

To examine how FABP5 knockdown suppressed cell proliferation, we next evaluated the effect of FABP5 knockdown on cell cycle and the apoptosis rate. Flow cytometry analyses demonstrated that G1 cell cycle arrest and apoptosis were induced by FABP5 suppression in PCa cells (Figure 6A, 6B, Supplementary Figure 6A and 6B).

FABP5 regulated metabolic gene expression via ERRα activation. To determine the molecular mechanisms underlying FABP5/ERRα-induced PCa proliferation, we analyzed energy metabolism associated with cell growth in PCa cells. Although many different types of cancer cells exhibit increased glycolysis, PCa is characterized by low glycolysis and altered fatty acid metabolism [17]. FABP5 is involved in intracellular fatty acid transport, and thus its depletion in PCa cells might lead to remodeling cancer metabolism. In addition, the genes transcriptionally regulated by FABP5/ERRα (as shown above) are associated with energy metabolism (oxidative phosphorylation, ATP5B; fatty acid oxidation, LCHAD; TCA cycle, ACO2, FH; mitochondrial dynamics, MFN2). Therefore, we examined the relationship between FABP5 expression level and the cellular energy status of PCa cells. Knockdown of FABP5 resulted in a significant increase in the AMP+ADP/ATP ratio (Figure 6C). In contrast, the intracellular ATP level was elevated in FABP5 overexpressing PNT2 cells (Figure 6D).

Because AMP levels were significantly increased in FABP5 knocked down PCa cells, we next examined the activation level of AMP-activated protein kinase (AMPK), a crucial cellular energy sensor. Immunoblot analyses revealed that levels of phosphorylated AMPK (Thr172), an active form of AMPK, were robustly increased by FABP5 knockdown (Figure 6E and Supplementary Figure 6C). Furthermore, its downstream target, forkhead box O3a (FOXO3A), a well-established tumor suppressor, was activated, along with increases in FOXO3A target genes (cell cycle inhibitor proteins p21 and p27 as well as pro-apoptotic protein p53 upregulated modulator of apoptosis (PUMA) (Figure 6F and Supplementary Figure 6D). In addition, an increase in the number of autophagosomes was observed in FABP5 knocked down PCa cells (Supplementary Figure 6E and 6F), confirming the reduction of energy production upon reduction of FABP5 in PCa cells. A recent study showed that AMPK regulated autophagy in normal mammalian cells [48]. These results suggest that the suppression of PCa cell proliferation induced by FABP5 knockdown may be attributed to an increase in AMP level followed by activation of the AMPK-FOXO3A signaling pathway.

Together our results show that FABP5 is a crucial regulator of altered metabolism in PCa cells, and thus development of a specific inhibitor for FABP5 as a molecular target would be a promising therapeutic strategy for PCa.

## DISCUSSION

Metabolic abnormalities have been observed in many cancer cells, and thus much attention has recently been paid to the studies on better understanding metabolic changes during carcinogenesis. Warburg first suggested that cancer cells preferentially use glycolysis as a major energy source even in the presence of oxygen and produce high levels of lactate and pyruvate [49, 50]. Metabolic reprogramming such as Warburg's effect and glutaminolysis has so far been accepted as a hallmark of cancer and a therapeutic target for cancer treatment [12, 13]. In addition, other studies recently revealed that lipid and energy metabolism also play a pivotal role in cancer progression and metastasis [14–16, 51, 52]. Thus, understanding metabolism in cancer cells is important for developing new therapeutic strategies and anticancer drugs. In fact, various compounds targeting metabolic enzymes have been used as selective drugs of neoplastic cells, such as methotrexate [53, 54].

Although previous studies showed that FABP5-PPARβ/δ signaling induced the expression of genes involved in cell growth and survival [34, 35, 55], we recently demonstrated that FABP5 regulates the expression of metabolic genes via a PPARβ/δ-independent pathway in colorectal cancer cells [31]. In the present study, we examined the effect of FABP5 on PPARβ/δ signaling using the PPARβ/δ agonist GW0742. In PCa cells, activation of PPARβ/δ with GW0742 resulted in an increase in the mRNA levels of ADRP, PDPK1 and ILK, a PPAR (β/δ and γ) target gene [56], while FABP5 expression level had no effect (Figure 3B, 3C and Supplementary Figure 3A). Interestingly, overexpression and knockdown of FABP5 in the GW0742-treated and -untreated cells had no effect in the expression of ADRP, PDPK1 and ILK, which phosphorylates AKT, leading to activation of survival signaling (Figure 3B, 3C and Supplementary Figure 3A). In addition, phosphorylated AKT were unchanged by knockdown of FABP5 (Figure 3D). These results are consistent with recent studies showing no changes in the phosphorylation of AKT in response to GW0742 in human HaCaT keratinocytes and colorectal cancer cells [31, 57–59]. These results show that FABP5 is not functionally associated with PPARβ/δ signaling pathway in PCa cells and suggest that FABP5 is associated with a novel signaling pathway other than PPARβ/δ to promote cancer cell growth and survival. Indeed, several studies showed that PPARβ/δ signaling does not potentiate the growth of human cancer cell lines and attenuates colon carcinogenesis [58–60]. FABP5 gene expression level did not significantly change in response to GW0742 (Figure 3B, 3C and Supplementary Figure 3A), suggesting that FABP5 gene expression is activated by a PPARβ/δ-independent signaling pathway in PCa cells. These results are contradictory to the report showing that FABP5 is involved in PPARβ/δ-dependent PCa cell growth [34]. A recent study reported that FABP5 promoted VEGF expression and angiogenesis through PPARγ during metastasis [61]. This result suggests that FABP5 functions as a fatty acid transporter for PPARγ to promote angiogenesis via VEGF gene expression.

**Figure 7:**
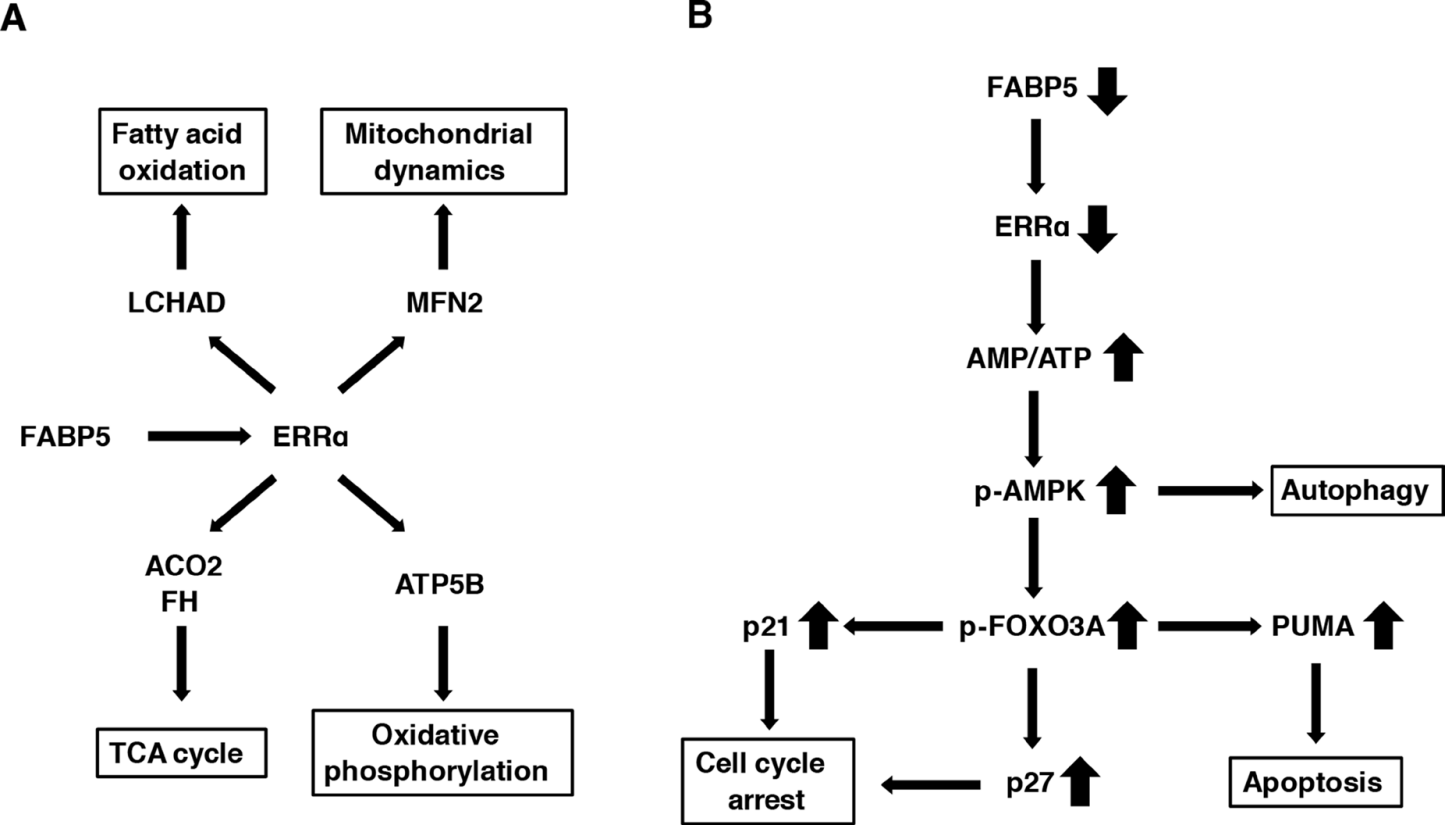
Schematic model for the FABP5-mediated transcriptional controlling network of mitochondrial functions in PCa cells (**A**) FABP5-ERRα crosstalk is required for mitochondrial functions and cell proliferation in PCa cells. FABP5 activated mRNA expression of more than one metabolic gene via ERRα signaling in PCa cells. (**B**) FABP5 knockdown induced high energy stress (increase of AMP/ATP ratio) in PCa cells. AMPK was activated by increased AMP/ATP ratio. Moreover, autophagy, apoptosis and cell cycle arrest were induced in PCa cells transfected with siRNA against FABP5 via the AMPK-FOXO3A signaling pathway. Thus, we considered that metabolic genes regulated by FABP5-ERRα played an essential role for energy homeostasis and viability in PCa cells. These results suggested that FABP5 may be a molecular target for the antitumorigenic treatments for PCa.

Thus, as the molecular mechanisms underlying the connection between high expression of FABP5 and cancer cell proliferation remains unclear, we searched for a novel crosstalk signaling with FABP5 in PCa cells. Our results show for the first time that FABP5 regulates energy metabolism via ERRα activation in PCa cells (Figure 7A). ERRs are members of the nuclear receptor superfamily consisting of ERRα, ERRβ and ERRγ. ERRs are considered orphan nuclear receptors, because the endogenous ligands for ERRs have not yet been identified [62]. Previous studies have shown that ERRs actually function in a constitutive manner [63]. However, several synthetic modulators for ERRs (such as the ERRα-specific inverse agonist XCT790) have been identified, suggesting that putative endogenous ligands or modulators would be present in cells. Indeed, co-regulatory proteins such as PGC-1α/PGC-1β and other cellular factors associated with ERRs have been reported [64]. However, characterization of these co-regulators in cancer cells has remained unclear.

ERRα and ERRγ play important roles in the regulation of various cellular metabolism pathways [65]. In addition, correlations have been found between the expression levels in ERRα/ERRγ and tumor progression in various cancers [66]. For example, ERRα is a nuclear receptor associated with mitochondrial functions and promotes tumorigenesis in various cancers [38–45]. Mitochondria are cytoplasmic organelles that regulate catabolism, anabolism and apoptosis [67]. In addition, mitochondria play a central role in fatty acid β-oxidation that leads to the release of energy and is associated with cell growth and metastasis in cancer cells [68]. Mitochondrial functions are aberrant in cancers and are targeted by drugs [69, 70]. Here, we revealed FABP5 that regulated metabolic gene expression (ATP5B, LCHAD, ACO2, FH and MFN2) and promoted energy metabolism in an ERRα-dependent, but not in PPARβ/δ-dependent manner (Figure 4 and Supplementary Figure 4B). Importantly, these ERRα target genes were induced in a PPAR-independent manner. Moreover, previous studies have shown that ERRα directly regulates cancer proliferation and migration [64, 66]. These results suggest that high expression of FABP5 cross talks with ERRα to promote cancer cell proliferation and metastasis. Further mechanistic studies are required to better understand FABP5 functions in cancer cells. Interestingly, ERRα regulates bone formation and transcriptional activation of prostate specific antigen [71–73]. PCa cells were characterized by a high rate (68%) of bone metastasis [74]. We speculate that these results might be associated with the findings that most of the PCa cells metastasize to the bone to be much more aggressive there.

Our results showed that FABP5 specifically interacted with ERRα and PGC-1β in the nucleus. PGC-1α/β is a co-activator for ERRα and PPARs, and regulates energy metabolism. PGC-1β was highly expressed whereas PGC-1α was not detectable in PCa cell lines (Supplementary Figure 4E). In addition, ERRα expression level was higher than that of PPAR in various PCa cell lines (Supplementary Figure 4E). Thus, we hypothesized that FABP5 preferentially functions in the ERRα/PGC-1β complex compared with PPARs in PCa cells. Importantly, the pull-down and immunoprecipitation assays demonstrated that FABP5 physically interacts with ERRα via the carboxyl terminal domain of ERRα containing the ligand-binding and AF-2 domains (Figure 5D). In the present study, we have clearly shown that FABP5 acts as a transcriptional cofactor for ERRα with PGC-1β to activate ERRα-target genes in the nucleus.

Our results also revealed an increase in AMP/ATP ratio in response to suppression of FABP5 and induction of apoptosis and cell cycle arrest through the AMPK-FOXO3A signaling pathway in PCa cells (Figure 7B), strongly suggesting that FABP5 regulates cell growth and mitochondrial functions that is essential for energy metabolism in PCa cells. The functional role of AMPK seems to be complex during carcinogenesis, because AMPK exhibits both tumor suppressor and oncogenic functions depending on the type of cancer [75, 76]. However, as FABP5 knockdown resulted in suppression of cell proliferation along with AMPK activation (T172 phosphorylation), AMPK seems to function as a tumor suppressor in PCa cells. Therefore, a FABP5-selective inhibitor or AICAR (AMPK-selective activator) might be a promising anti-tumorigenic reagent for PCa cells. Thus, the present study clearly shows that FABP5 plays an important role in regulating the activity of AMPK that is a sensor of cellular energy status directing metabolic adaptation to support cellular proliferation and survival in PCa cells.

Here we have demonstrated that FABP5 activated the expression of metabolic genes (ATP5B, LCHAD, ACO2, FH and MFN2) in an ERRα-dependent manner in highly aggressive PCa cells. Importantly, these ERRα target genes were not induced by PPAR. These results strongly suggest that a novel FABP5-ERRα signaling axis plays an important role in the cell proliferation and metastatic potential in PCa cells.

## MATERIALS AND METHODS

### Reagents

GW0742 was purchased from Focus Biomolecules. Oleic acid, Palmitic acid and Stearic acid were purchased from Sigma-Aldrich. DAUDA (a fluorescent analog of fatty acid:11–(Dansylamino)undecanoic acid) was purchased from Santa Cruz Biotechnology. Antibodies were obtained as follow: Cell Signaling Technology (Rabbit anti-FABP5 (Cat# 39926), Rabbit anti-AMPKα (Cat# 5832S), Rabbit anti-p-AMPKα (T172)(Cat# 2535), Rabbit anti-FOXO3A (Cat# 2497S), Rabbit anti-p-FOXO3A (S413)(Cat# 8174S), Rabbit anti-p27 (Cat# 3686S), Rabbit anti-Puma (Cat# 12450), Rabbit anti-ERRα (Cat# 13826), Rabbit anti-AKT (Cat# 4691), Rabbit anti-p-AKT (S473)(Cat# 4060)), Santa Cruz Biotechnology (Rabbit anti-MAPK15 (Cat# sc-130814), Rabbit anti-p21(Cat# sc-397), Mouse anti-β-actin (Cat# sc-47778), Mouse anti-α-tubulin (Cat# sc-8035), Rabbit anti-TBP (Cat# sc-204), Nomal rabbit-IgG (Cat# sc-2027)), MBL (Rabbit anti-GST-tag HRP-DirecT (Cat# PM013-7), Rabbit anti-DDDDK-tag HRP-DirecT (Cat# PM020-7), Rabbit anti-DDDDK-tag-Agarose (Cat# PM020-8), Rabbit anti-DDDDK-tag mAb (Cat#M185-3S)), Bethyl Laboratories (Rabbit anti-PGC-1β (Cat# A302-274A)), Enzo Life Science (HRP-conjugated goat anti-mouse IgG, HRP-conjugated goat anti-rabbit IgG), BD Biosciences (Rabbit anti-active caspase-3 (Cat# 559565)), Jackson ImmunoResearch (FITC-conjugated Donkey anti-rabbit IgG (Cat# 711-096-152)), Abcam (Alexa Fluor^®^ 488-conjugated donkey anti-rabbit IgG H&G (Cat# ab150073), Alexa Fluor^®^ 647-conjugated goat anti-mouse IgG H&G (Abcam, ab150115)).

### Cell lines

Human prostate cancer cells (PC-3, DU-145 and LNCaP) and 293T cells were obtained from RIKEN Bio Resource Center. Other human prostate cancer cells (22Rv1 and RWPE-1) were obtained from American Type Culture Collection (ATCC). PC-3M (human prostate cancer cell lines) and normal prostate cell lines (PNT2) were generous gifts from Dr. Youqiang Ke (Liverpool University, UK). All cell lines were male and cultured at 37°C in a humidified atmosphere (5% CO_2_). All cell lines, except for 293T, were grown in RPMI 1640 medium (Thermo Scientific) supplemented with 10% (v/v) FBS (Thermo Scientific). The 293T cell line was grown in DMEM/high glucose (Thermo Scientific) supplemented with 10% (v/v) FBS (Thermo Scientific). All cells tested negative for mycoplasma.

### Plasmids

The human FABP5 cDNA was amplified from PC-3 cells and subcloned into the pCI-neo mammalian expression vector (Promega). Human ERRα was amplified from PC-3 cells and subcloned into the pGEX-2T GST Expression Vector (GE Healthcare). The NES-FABP5 construct was produced by inserting an NES (nuclear export signal) sequence into pCI-neo/FABP5. LC3B was amplified from PC-3 cells and subcloned into the pTurboGFP-N vector (Evrogen Joint Stock Company). The pGL3-basic and pGL4.74 [hRluc/TK] vectors were purchased from Promega. The human ATP5B promoter region was amplified from PC-3 cells by PCR. Mutated luciferase reporter plasmids were generated by the KOD-plus-mutagenesis kit (Toyobo). PCR primers for ATP5B (−300–+108) and mutagenesis (ΔERRE) were conducted as previously reported [36] and shown in primers section of Materials and Methods.

### siRNA

Human prostate cancer cell lines (DU-145, PC-3 and PC-3M) were transfected with 20 nM negative control siRNA or siRNA against FABP5 or PPARβ/δ using Lipofectamine™ 2000 (Life Technologies) according to the manufacturer's instructions. The mRNA expression levels were measured at 72 h after transfection by qPCR. All siRNAs were purchased from IDT (FABP5 siRNA: HSC.RNAI.N001444.12.1, FABP5 siRNA-2: HSC.RNAI.N001444.12.2, PPARβ/δ siRNA: HSC.RNAI.N177435.12.2, PPARβ/δ siRNA-2: HSC.RNAI.N001171818.12.8, ERRα siRNA: HSC.RNAI.N004451.12.2, ERRα siRNA-2: HSC.RNAI.N004451.12.4).

### qPCR

Total RNA was extracted using the TRI Reagent (Molecular Research Center), and cDNAs were synthesized from 500 ng of total RNA using the Rever-Tra Ace qPCR RT Master Mix (Toyobo) according to the manufacturer's instructions. Quantitative real-time PCR (qPCR) analyses were performed with the Thermal Cycler Dice Real Time system (TaKaRa) using THUNDERBIRD SYBR qPCR Mix (Toyobo) according to the manufacturer's instructions. Primers used for qPCR are shown in primers section.

### Western blot analysis

Cells were lysed using RIPA buffer with protease inhibitor cocktail (Nacalai Tesque). Protein samples (30 μg) were fractionated by SDS-PAGE using 8% SDS-polyacrylamide gels. Immunoblotting was carried out using the appropriate antibodies and Pierce Western Blotting Substrate (Thermo Scientific) according to the manufacturer's instructions. Chemiluminescent signals were captured by the Image Quant LAS4000 Mini (GE Healthcare Biosciences).

### Cell proliferation assay

DU-145, PC-3 and PC-3M cells were plated in six-well plates and transfected with control siRNA, siRNA against FABP5, PPARβ/δ or pCI-neo/NES-FABP5 or treated with GSK-3787. Cells were counted at 24, 48, and 72 h after transfection or treatment. PNT2 cells were plated in six-well plates and transfected with pCI-neo (control), pCI-neo/FABP5 or pCI-neo/NES-FABP5. Cells were counted at 24, 48, 72, 96 and 120 h after transfection.

### Immunostaining and fluorescence microscopy

Cells transfected with vector, pCI-neo/FABP5 or pCI-neo/NES-FABP5 were fixed with 4% PFA for 20 min (Nacalai Tesque). Cells were incubated with the following primary antibodies overnight at 4°C: anti-FABP5 (1:100) (Cell Signaling Technology), anti-α-tubulin (1:1000) (Santa Cruz Biotechnology), and anti-ERRα (1:100) (Cell Signaling Technology). After washing with TBS twice, cells were incubated with the following secondary antibodies overnight at 4°C: Alexa Fluor^®^ 488-conjugated donkey anti-rabbit IgG H&G (1:2000) (Abcam) and Alexa Fluor^®^ 647-conjugated goat anti-mouse IgG H&G (1:2000) (Abcam). Cells were stained with Hoechst 33342 (Nacalai Tesque). Laser scanning confocal microscopy was performed on an Olympus FV1000.

### Luciferase assay

Luciferase assay was performed using the Dual-Luciferase Reporter Assay Kit (Promega) according to the manufacturer's instructions. The luciferase reporter contained the ATP5B promoter region with the wild-type (WT) sequence or mutant sequence deleted for ERRE(CCAAGGACA)(ΔERRE). Site-directed mutagenesis was performed using the pGL3-ATP5B/−300~+108 construct as a template. Cells were seeded into 24-well plates and transfected with expression vectors and siRNA using Lipofectamine™ 2000 (Life Technologies). The pGL4.74 [hRluc/TK] vector (Promega) was used as an internal control for transfection efficiency. Luciferase activity was measured on a PowerScan HT microplate reader (BioTek).

### Nucleocytoplasmic fractionation

Cells (1 × 10^7^) were collected by scraping from culture dishes and washed twice with cold PBS. Cells were resuspended in hypotonic buffer (20 mM Tris-HCl, pH 7.4, 10 mM NaCl, 3 mM MgCl_2_) (Nacalai Tesque) and incubated on ice for 15 min. Cells were gently homogenized and then centrifuged for 10 min at 3,000 rpm at 4°C. The supernatants that contain the cytoplasmic fraction were transferred and saved as samples for SDS-PAGE. The pellets that contain the nuclear fraction were resuspended in lysis buffer and centrifuged for 30 min at 15,000 rpm at 4°C. Supernatants were transferred to a clean microtubes.

### Chromatin immunoprecipitation (ChIP) assay

ChIP assay was performed using the ChIP-IT Express Enzymatic kit (Active Motif) according to the manufacturer's instructions. This kit contained almost all reagents for this assay (including lysis buffer, without antibody). Cells (1 × 10^7^) were fixed by 37% formaldehyde and used as samples for ChIP. The antibody against FLAG tag was purchased from MBL. qPCR analyses were performed with the Thermal Cycler Dice Real Time system (TaKaRa) using THUNDERBIRD^®^ SYBR^®^ qPCR Mix (Toyobo). Primers used for qPCR are shown in primers section.

### GST pull-down assay

The pGEX-2T/ERRα wt, Δ300aa, Δ398aa or pGEX-2T vector (GE Healthcare) was transformed into the BL21 strain (RBC Bioscience) and GST fusion protein expression was induced with 0.1 mM of IPTG (Nacalai Tesque). GST or GST-ERRα was purified using GSTrap™ FF (GE Healthcare). Whole cell lysates were incubated with GST-Accept (Nacalai Tesque) overnight at 4°C. Complexes were washed three times with washing buffer (50 mM Tris-HCl, pH 7.5) (Nacalai Tesque), 150 mM NaCl (Nacalai Tesque), 0.05% Tween20 (Wako)). Proteins interacting with ERRα were eluted with the sample buffer (2% SDS, 3% glycerol, 125 mM Tris-HCl, pH 6.5), 4% 2-mercaptoethanol, and 0.0125% BPB (all from Nacalai Tesque) and detected by western blot analysis.

### Immunoprecipitation

Cells were lysed using lysis buffer (50 mM Tris-HCl, 150 mM NaCl, 1mM EDTA, 1% NP-40, protease inhibitor cocktail) (all reagents were purchased from Nacalai Tesque). Whole cell lysates were incubated with agarose-conjugated anti-DDDDK-Tag (MBL) overnight at 4°C. The immunoprecipitated complexes were washed three times with washing buffer. Proteins were eluted by boiling in sample buffer and then processed for western blot analysis.

### Cell cycle analysis

Cell cycle distribution was measured by flow cytometry. Collected cells were washed with ice-cold PBS and fixed with 70% ethanol, followed by treatment with stain solution (1% Triton X-100 (Nacalai Tesque), 50 μg/mL propidium iodide (SIGMA), 40 mM sodium citrate (Wako), 100 μg/mL RNase A (Wako)) for 1 h at room temperature in the dark. Cells were then analyzed using a FACSCalibur flow cytometer (BD Biosciences) and data were analyzed using the CellQuest software (BD Biosciences).

### Apoptosis assay

Cells transfected with negative control siRNA or FABP5 siRNA were collected and fixed with BD Cytofix/Cytoperm Fixation and Permeabilization Solution (BD Biosciences) for 20 min at 4°C in the dark. Cells were then washed with washing buffer and incubated with anti-active caspase-3 antibody (BD Biosciences) for 1.5 h at room temperature. After washing with washing buffer, the cells were then incubated with FITC-conjugated anti-rabbit IgG (Jackson ImmunoResearch) for 1 h at room temperature in the dark and diluted in PBS. The cells were analyzed using a FACSCalibur flow cytometer (BD Biosciences) and the CellQuest software (BD Biosciences).

### Autophagy detection assay

Autophagy was measured by changes in TurboGFP-LC3B localization. LC3B interacts with the autophagosome membrane at an early stage of autophagy. TurboGFP-LC3B punctate structures were counted on an EVOS^®^ FL Auto Cell Imaging System (Thermo Scientific). Boxplots were created using BoxPlotR. Boxplots showed the number of punctate pCI-neo/TurboGFP-LC3B in control siRNA or siFABP5-transfected PC-3 cells 72 h after transfection.

### Quantification of AMP, ADP and ATP

Cellular ATP was extracted and measured using the IntraCellular ATP assay kit (Toyo-ink), according to the manufacturer's protocols. Cellular AMP (+ADP) was extracted and measured using the AMP-Glo™ Assay kit (Promega), according to the manufacturer's protocols.

### Fatty acid-binding assay for NES-FABP5

Defatted FABP5 and NES-FABP5 proteins were titrated with DAUDA (a fluorescent analog of fatty acid: 11–(dansylamino)undecanoic acid) dissolved in ethanol. Fluorescence intensity at 550 nm during excitation at 335 nm was measured on a PowerScan HT microplate reader (BioTek) at 25°C. Fluorescence readings were corrected for DAUDA fluorescence without protein. Affinity for fatty acids was estimated by displacement of DAUDA by the added fatty acids [77]. FABP5 and NES-FABP5 (1.5 μM) were mixed with 1.25 μM of DAUDA, and then increasing amounts of fatty acids dissolved in ethanol were added. The decrease in fluorescence intensity of each sample was recorded.

### Primers

Primers were synthesized by Integrated DNA Technologies (IDT). Primer: ATP5B promoter (−300~+108), sense 5′-GGACTCGAGGCCCCTATGGCT GTCACCTAG-3′, antisense 5′-ATGCCATGGCGTAGT CCGGGTGGA-3′. Primer: Mutagenesis (ΔERRE), sense

5′-GGCAAAGACAGGCCACGCAC-3′, antisense 5′-GCTTTCTCCTTAGATAGGTC-3′. Primer: 18S rRNA, sense 5′-CGGCTACCACATCCAAGGAA-3′, antisense 5′-GCTGGAATTACCGCGGCT-3′. Primer: FABP5, sense 5′-GCTGATGGCAGAAAAACTCAGA -3′, antisense 5′-CCTGATGCTGAACCAATGCA-3′. Primer: ERRα, sense 5′-TGTCAATTCAGACTCTGTGC-3′, antisense 5′-CCAGCTTCACCCCATAGAAA-3′. Primer: LCHAD, sense 5′-GGACCAGGACTAAAACCTCCA-3′, antisense 5′-AGTCATGGCATACGCTGTCA-3′. Primer: ACO2, sense 5′-GCCCAACGAGTACATCCATT-3′, antisense 5′-GACTTGCCTCGCTCAATTTC-3′. Primer: FH, sense 5′-GCATCCCAACGATCATGTTA-3′, antisense 5′-GGATTTTGCATCAAGAGCATC-3′. Primer: MFN2, sense 5′-GGATGCTGATGTGTTTGTGC-3′, antisense 5′-AGCGGTTGTTCAGGATGAAG-3′. Primer: PPARα, sense 5′-AGGCTATCATTACGGAGTCCAC-3′, antisense 5′-GTCGCACTTGTCATACACCAG-3′. Primer: PPARβ/δ, sense 5′-AGTGACCTGGCCCTATTCATT-3′, antisense 5′-TTGGCCTGCAGGTGGAATTC-3′. Primer: PPARγ1, sense 5′-GACCACTCCCACTCCTTTGA-3′, antisense 5′-CGACATTCAATTGCCATGAG-3′. Primer: PPARγ2, sense 5′-TCCATGCTGTTATGGGTGAA-3′, antisense 5′-GTGTCAACCATGGTCATTTCTT-3′. Primer: ERRβ, sense 5′-CATGAAATGCCTCAAAGTGG -3′, antisense 5′-AGGTATGGGCTGCTCTCTGA-3′. Primer: ERRγ, sense 5′-CAGTGACATCAAAGCCCTC A-3′, antisense 5′-TTCCATCCAAGCACTCTGC-3′. Primer: PGC-1α, sense 5′-TTCTCGACACAGGT CGTGTT-3′, antisense 5′-CGGTGTCTGTAGTGGCTTG A-3′. Primer: PGC-1β, sense 5′-CCCTTCTCCTGTTCCT TTGGA-3′, antisense 5′-CCTTTGGAGGACGC CTTCT-3′. Primer: Chip ATP5B, sense 5′-GCCCCTATGGCTGTCACCTA-3′, antisense 5′-TCCTGTTGAAAGTGCGTGGC-3′. Primer: FABP1, sense 5′-GCAGAGCCAGGAAAACTTTG-3′, antisense 5′-TCTCCCCTGTCATTGTCTCC-3′. Primer: FABP2, sense 5′-TTGGAAGGTAGACCGGAGTG-3′, antisense 5′-AGGTCCCCCTGAGTTCAGTT-3′. Primer: FABP3, sense 5′- AGCCTAGCCCAGCATCACTA-3′, antisense 5′-GTCCCCATTCTTTTCGATGA-3′. Primer: FABP4, sense 5′-TACTGGGCCAGGAATTTGAC-3′, antisense 5′-GTGGAAGTGACGCCTTTCAT-3′. Primer: FABP6, sense 5′-CTCATCCCTCTGCTCTCTGG-3′, antisense 5′-GTGCTGGGACCAAGTGAAGT-3′. Primer: FABP7, sense 5′-GTGGGAAATGTGACCAAACC-3′, antisense 5′-CTTTGCCATCCCATTTCTGT-3′. Primer: FABP8, sense 5′-CAAGCTAGGCCAGGAATTTG-3′, antisense 5′-CCACGCCCTTCATTTTACAT-3′. Primer: FABP9, sense 5′-TGATGGGAAAATGATGACCA-3′, antisense 5′-TCTCTTTGCCAAGCCATTTT-3′. Primer: FABP12, sense 5′-GATGGGAAAATGGTGGTGGA-3′, antisense 5′-TTGATGATACTTTCTCGTATGTTCG-3′. Primer: ILK, sense 5′-AAGGTGCTGAAGGTTCGAGA-3′, antisense 5′-ATACGGCATCCAGTGTGTGA-3′.

### Quantification and statistical analysis

Data were expressed as the means ± standard deviation (S.D.) calculated with GraphPad Prism software (version 7.03, GraphPad Software Inc.). Exact value of N and what N represents for each experiment are annotated in each figure legend. Statistical analysis was performed using the unpaired two-tailed *t*-test to determine the significance of differences between two groups. For multiple comparisons, one-way analysis of variance (ANOVA) with Dunnett's or Tukey's post-hoc test or two-way ANOVA with Tukey's post-hoc test was used. A *p*-value less than 0.05 was considered statistically significant, and indicated by the following indications: ^*^P < 0.05, ^**^P < 0.01.

## SUPPLEMENTARY MATERIALS FIGURES



## Abbreviations

FABP5: fatty acid-binding protein 5
ERRα: estrogen-related receptor α
ATP5B: ATP synthase subunit beta
LCHAD: long-chain 3-hydroxyacyl-CoA dehydrogenase
ACO2: aconitase 2
FH: fumarate hydratase
MFN2: mitofusin 2
PPARβ/δ: peroxisome-proliferator-activated receptor β/δ
ADRP: adipose differentiation-related protein
PDPK1: 3-phosphoinositide-dependent protein kinase-1
ERK8: extracellular signal-regulated kinase 8
PGC-1β: PPAR gamma co-activator 1β
PUMA: p53 up-regulated modulator of apoptosis
FOXO3A: forkhead box O3a
AMPK: AMP-activated protein kinase.
