# Supplementary material for: A novel fatty acid-binding protein 5-estrogen-related receptor α signaling pathway promotes cell growth and energy metabolism in prostate cancer cells
Source: Oncotarget. 2018 Aug 3;9(60):31753–70. doi: 10.18632/oncotarget.25878 (PMC6114981; doi:10.18632/oncotarget.25878)
Supplement: Supplementary file 1 [file oncotarget-09-31753-s001.pdf]

A novel fatty acid-binding protein 5-estrogen-related receptor α signaling pathway promotes cell growth and energy metabolism in prostate cancer cells

SUPPLEMENTARY MATERIALS

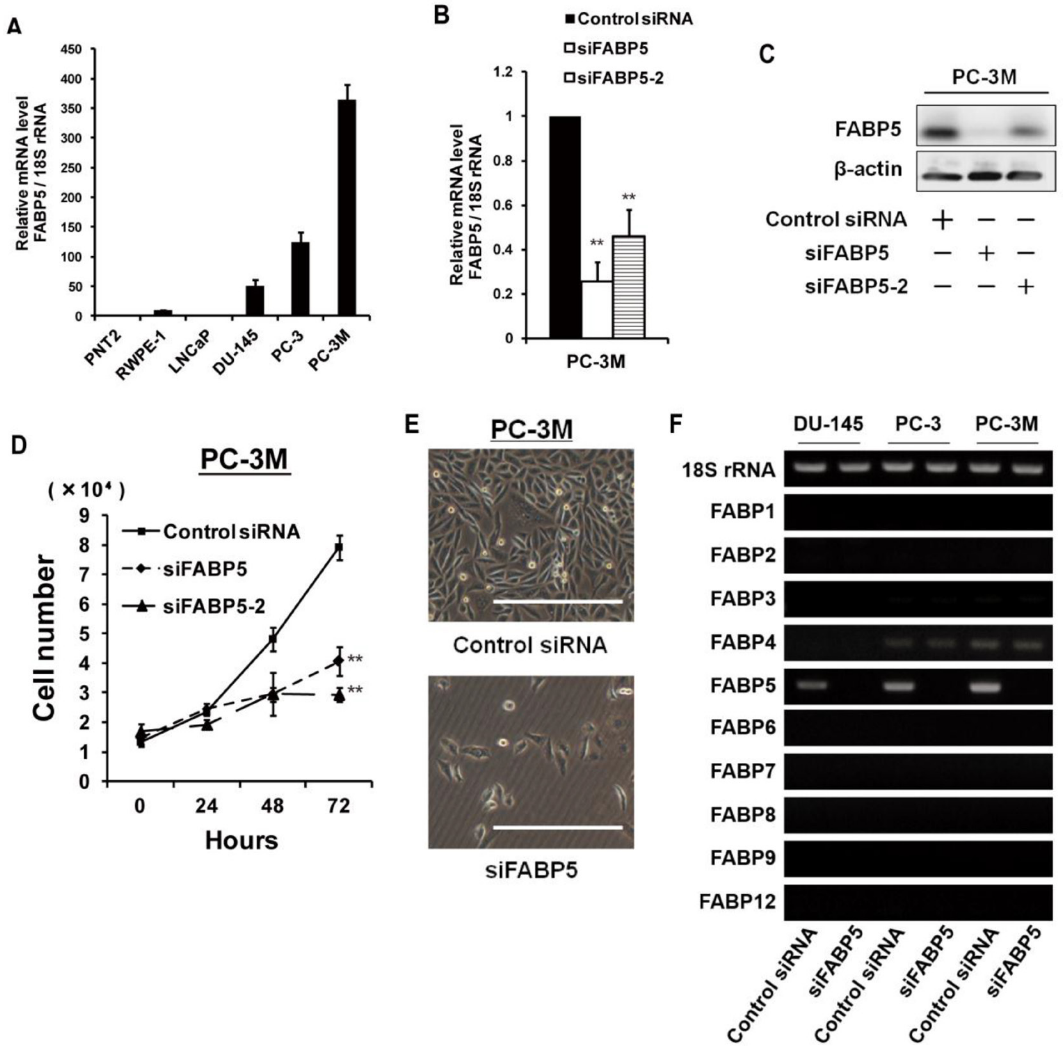

Supplementary Figure 1: FABP5 promotes cell growth in PC-3M cells. Related to Figure 1. (**A**) Measurement of levels of FABP5 expression in prostate benign and cancer cell lines by qPCR. (**B**) Relative levels of FABP5 mRNA in control siRNA or siFABP5 transfected PC-3M by qPCR. $^{**}P < 0.01$. (**C**) Detection of FABP5 in control siRNA or siFABP5 transfected PC-3M by western blot analysis. β-actin was used as the endogenous control. Results shown are representative of three independent experiments. (**D**) Cell growth of control siRNA or siFABP5 transfected PC-3M. Cells were counted at the indicated times. Results are means ± S.D. for three independent experiments. $^{**}P < 0.01$. (**E**) Microscopy of cells transfected with control siRNA (upper) or siRNA targeting FABP5 (lower) 72 h after transfection. Scale bar, 500 µm. (**F**) mRNA expression levels of FABP family were measured by semi-quantitative PCR in prostate cancer cell lines transfected with control siRNA or siRNA targeting FABP5. Results shown are representative of three independent experiments.

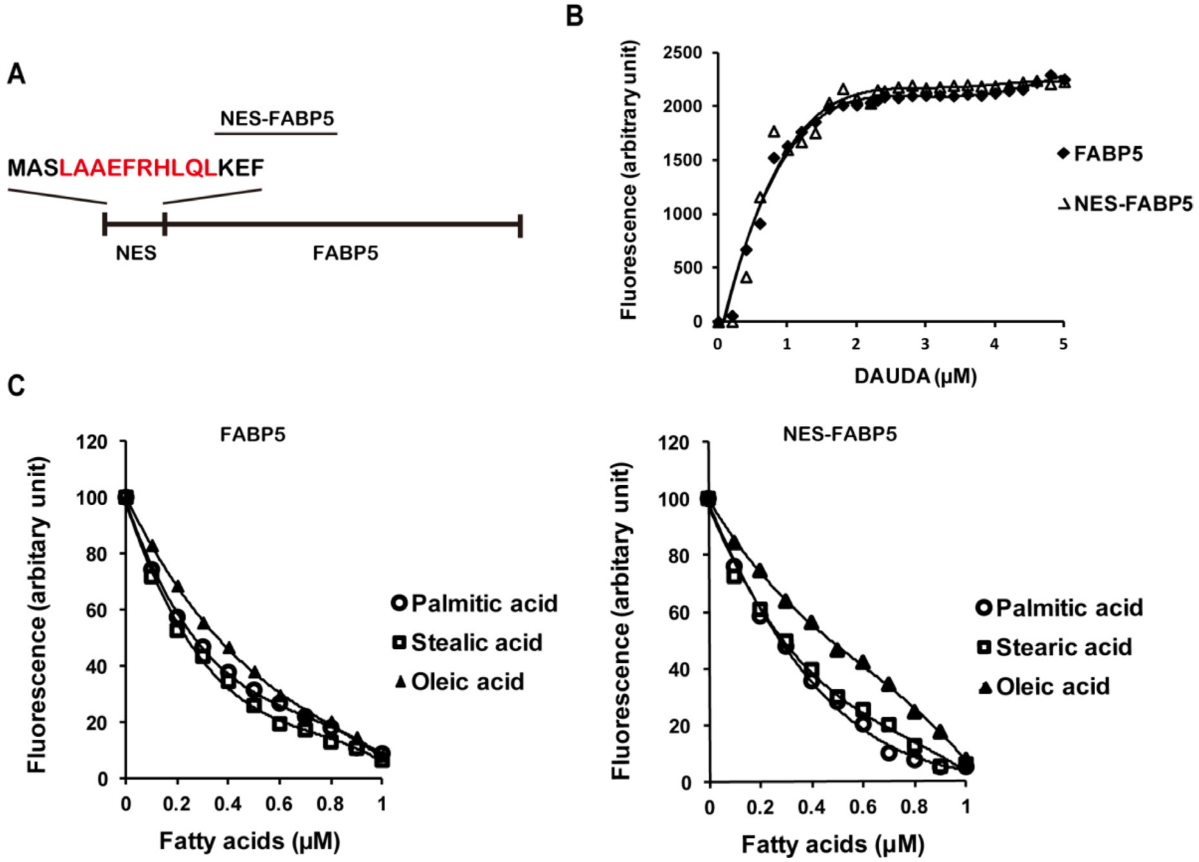

Supplementary Figure 2: NES-FABP5 binds to fatty acids with a high affinity similar to that found in the wild type FABP5. Related to Figure 2. (**A**) Schematic structure of NES-FABP5. Nuclear export signal (NES) is determined by reference to STAT1. (**B**) Fatty acid binding assay. FABP5 or NES-FABP5 proteins was titrated with DAUDA dissolved in ethanol. Fluorescence intensity at 550 nm during excitation at 335 nm was measured on a PowerScan HT microplate reader (BioTek) at 25° C. Fluorescence readings were corrected for DAUDA fluorescence without protein. (**C**) Affinity for fatty acids was estimated by displacement of DAUDA by the added fatty acids. FABP5 or NES-FABP5 (1.5 µM) was mixed with 1.25 µM of DAUDA, and then increasing amount of fatty acid (palmitic acid, stearic acid or oleic acid) dissolved in ethanol was added to it. Decrease in fluorescence intensity of FABP5 was recorded and compared with that in NES-FABP5.

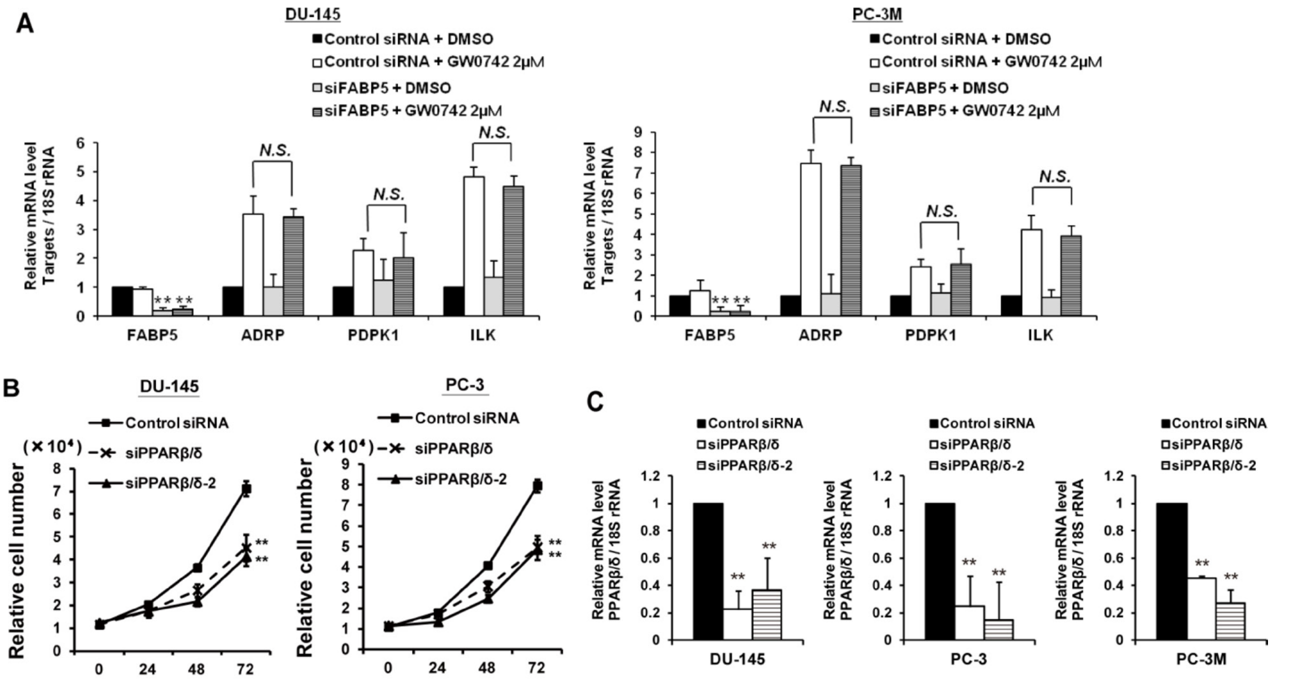

Supplementary Figure 3: The contribution of FABP5-PPARβ/δ crosstalk to cell gowth in PCa cells. Related to Figure3. (**A**) FABP5, ADRP, PDPK1 and ILK mRNA expression levels in DU-145 and PC-3M transfected control siRNA or siFABP5 and treated with DMSO or GW0742. Relative mRNA levels were measured by qPCR. Results are means ± S.D. for three independent experiments. (**B**) Cell growth of control siRNA or siPPARβ/δ transfected PCa cells. Cells were counted at the indicated times. $^{**}P < 0.01$. (**C**) PPARβ/δ mRNA expression levels in siRNA against PPARβ/δ transfected prostate cancer cells. Relative mRNA levels were measured by qPCR. Results are means ± S.D. for three independent experiments. $^{**}P < 0.01$.

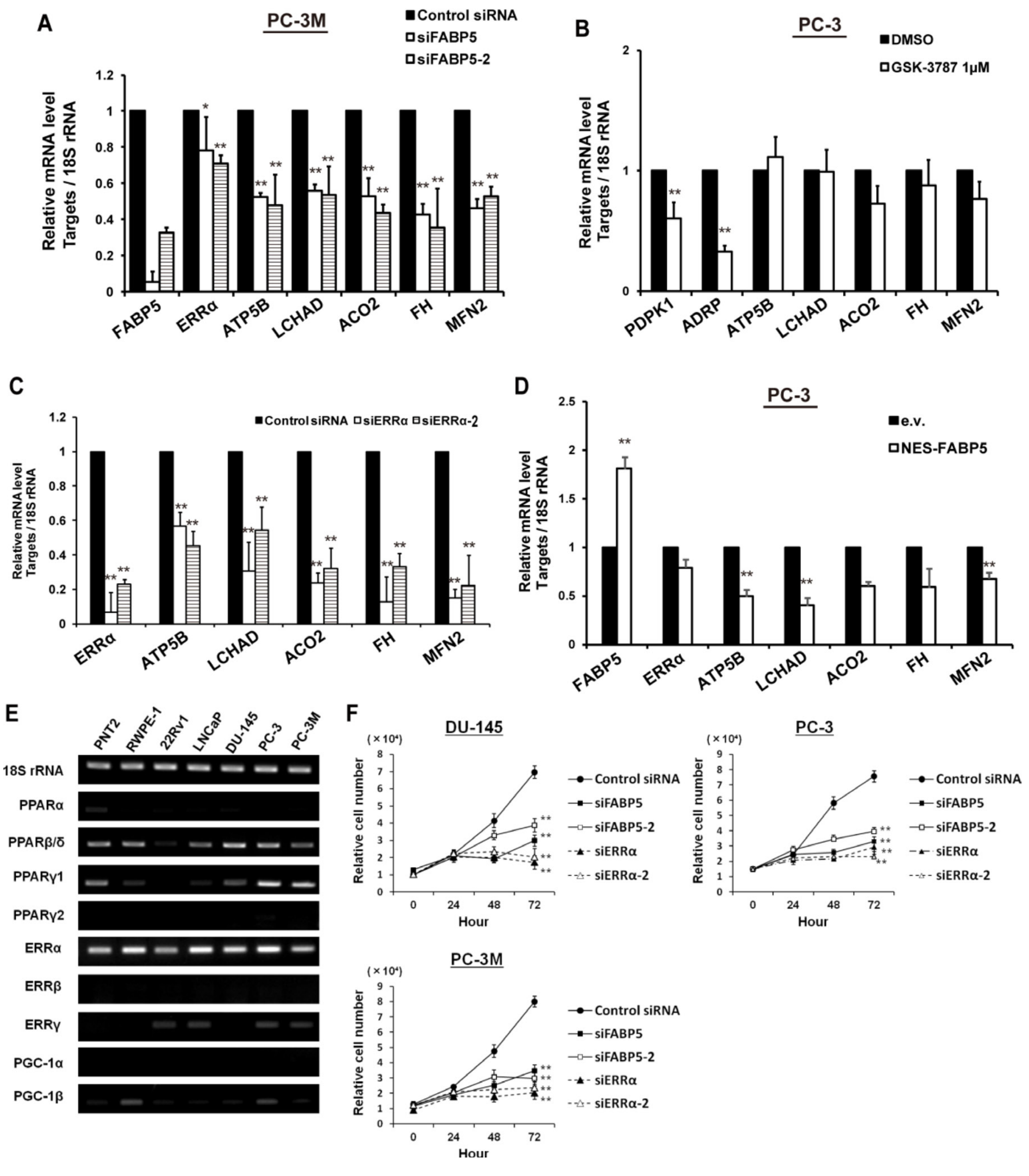

Supplementary Figure 4: ERRα target genes are up-regulated by FABP5 in PCa cells. Related to Figure 4. (**A**) PC-3M cells were transfected with control siRNA or siFABP5. mRNA levels were determined by qPCR. Results are means ± S.D. for three independent experiments. $^{**}P < 0.01$. $^{*}P < 0.05$. (**B**) mRNA expression levels of ERRα target genes were measured by qPCR in PPARβ/δ antagonist (GSK-3787) treated PC-3. PDPK1 and ADRP were PPARβ/δ direct target genes. Results are means ± S.D. for three independent experiments. $^{**}P < 0.01$. (**C**) mRNA expression levels of ERRα target genes were measured by siRNA against ERRα transfected PC-3. Results are means ± S.D. for three independent experiments. $^{**}P < 0.01$. (**D**) PC-3 cells were transfected with pCI-neo/NES-FABP5. mRNA levels of ERRα target genes were measured by qPCR. Results are means ± S.D. for three independent experiments. $^{**}P < 0.01$. (**E**) mRNA expression levels of PPARs, ERRs and PGC-1s were measured by semi-quantitative PCR in prostate cancer cell lines. Results are means ± S.D. for three independent experiments. Results shown are representative of three independent experiments. (**F**) Cell growth of control siRNA or siFABP5 or siERRα transfected DU-145, PC-3 and PC-3M. Cells were counted at the indicated times. Results are means ± S.D. for three independent experiments. $^{**}P < 0.01$.

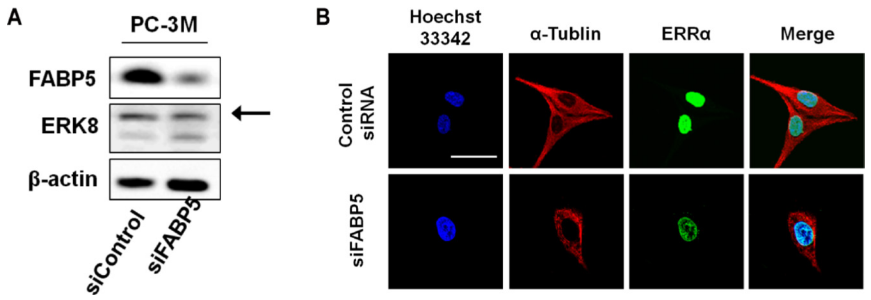

Supplementary Figure 5: Intracellular localization of ERRαin PCa cells transfected with siRNA against FABP5 (siFABP5). Related to Figure 5. (**A**) Detection of ERK8 expression level in PC-3M cells transfected with control siRNA or siFABP5 by western blot analysis. The arrow indicated ERK8 band. Results shown are representative of three independent experiments. (**B**) Intracellular localization of ERRα in DU-145 cells transfected with control siRNA or siFABP5. Results shown are representative of three independent experiments. Scale bar, 50 μm.

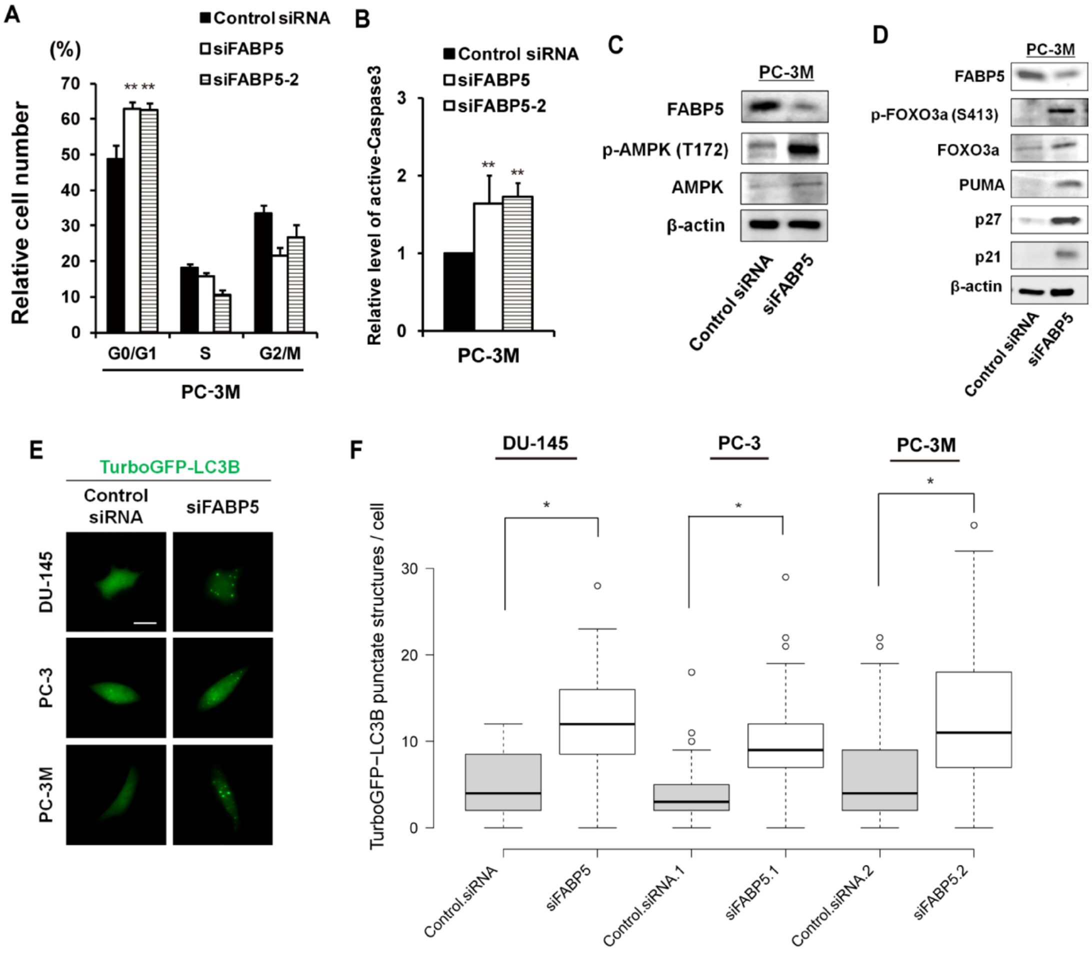

Supplementary Figure 6: FABP5 knockdown induces G1 cell cycle arrest, apoptosis and autophagy in PCa cells. Related to Figure 6. (**A**) Cell cycle analysis of control siRNA (20 nM) or siFABP5 (20 nM) transfected PC-3M by FCM. Cell-cycle distribution is represented as the percentage of cells at each phase. $^{**}P < 0.01$. (**B**) Apoptosis analysis. Apoptosis was measured by cleaved caspase 3 (active form) level by Flow cytometry. $^{**}P < 0.01$. (**C**) Western blot analysis of AMPK and phosphorylated AMPK. PC-3M cells were transfected with control siRNA or siFABP5. Results shown are representative of three independent experiments. (**D**) Protein expression levels of FOXO3a and its target genes were detected by western blot analysis. PC-3M cells were transfected with control siRNA or siFABP5. Results shown are representative of three independent experiments. (**E**) Microscopy of punctate pCI-neo/TurboGFP-LC3B in control siRNA or siFABP5 transfected PC-3 cells 72 h after transfection. (**F**) Boxplot of the number of punctate pCI-neo/TurboGFP-LC3B in control siRNA or siFABP5 transfected PC-3 cells 72 h after transfection. Center lines showed the medians; box limits indicated the 25th and 75th percentiles as determined by R software. whiskers extend 1.5 times the interquartile range from the 25th and 75th percentiles. outliers are represented by dots (data points were plotted as open circles.). $^{**}P < 0.05$ (n = 100).
